# Structural Variation among Wild and Industrial Strains of *Penicillium chrysogenum*

**DOI:** 10.1371/journal.pone.0096784

**Published:** 2014-05-13

**Authors:** Valerie L. Wong, Christopher E. Ellison, Michael B. Eisen, Lior Pachter, Rachel B. Brem

**Affiliations:** 1 Department of Plant and Microbial Biology, University of California Berkeley, Berkeley, California, United States of America; 2 Department of Molecular and Cell Biology, University of California Berkeley, Berkeley, California, United States of America; 3 Departments of Mathematics, Molecular and Cell Biology, and Electrical Engineering and Computer Science, University of California Berkeley, Berkeley, California, United States of America; Wilfrid Laurier University, Canada

## Abstract

Strain selection and strain improvement are the first, and arguably most important, steps in the industrial production of biological compounds by microorganisms. While traditional methods of mutagenesis and selection have been effective in improving production of compounds at a commercial scale, the genetic changes underpinning the altered phenotypes have remained largely unclear. We utilized high-throughput Illumina short read sequencing of a wild *Penicillium chrysogenum* strain in order to make whole genome comparisons to a sequenced improved strain (WIS 54–1255). We developed an assembly-free method of identifying chromosomal rearrangements and validated the *in silico* predictions with a PCR-based assay and Sanger sequencing. Despite many rounds of mutagen treatment and artificial selection, WIS 54–1255 differs from its wild progenitor at only one of the identified rearrangements. We suggest that natural variants predisposed for high penicillin production were instrumental in the success of WIS 54–1255 as an industrial strain. In addition to finding a previously published inversion in the penicillin biosynthesis cluster, we located several genes related to penicillin production associated with these rearrangements. By comparing the configuration of rearrangement events among several historically important strains known to be high penicillin producers to a collection of recently isolated wild strains, we suggest that wild strains with rearrangements similar to those in known high penicillin producers may be viable candidates for further improvement efforts.

## Introduction

The discovery of penicillin and its antibiotic properties begun by Alexander Fleming and developed by Chain and Florey was a landmark in medicine and pharmacology [1], [2]. However, Fleming's original strain produced only small quantities of penicillin. The efforts to make antibiotics more available, particularly in response to great demand during World War II, entailed both a search for wild strains with enhanced production of penicillin and improvement of strains already in culture [3]. Notably, Raper, Alexander, and Coghill [4] cultivated and tested isolates from a variety of food products, spoiled produce, and soils. Nearly all of their *Penicillium* strains produced detectable levels of penicillin, but very few were comparable to the best industrially important strains of the time [4]. The new isolates formed a bimodal distribution of penicillin production [4], indicative of natural variation in the wild population and suggesting that some wild strains may be predisposed to be high penicillin producers and to give rise to viable industrial strains.

Many commercial strains used by pharmaceutical companies such as Lilly Industries and Wyeth Lab trace their ancestry back to a single wild strain (*P. chrysogenum* NRRL 1951) isolated from a moldy cantaloupe found in Peoria, Illinois [3], [5]–[7]. Compared to its improved progeny, NRRL 1951 is a relatively low penicillin producer [4]. Multiple rounds of non-directed mutagenesis and selection led to numerous sub-lineages, including the well-studied Wisconsin 54–1255, and later industrial strains with vastly higher production levels [8]. Despite decades of work on strain improvement, little is known about the indirect regulation of penicillin biosynthesis and how improvement occurred in this “Wisconsin family” of strains. With the recent availability of high-throughput DNA sequencing, it is now possible to compare whole genomes of wild and industrial strains in order to identify genomic differences that may be responsible for the improved phenotype [9].

The *P. chrysogenum* core genes for penicillin biosynthesis (*pcbAB, pcbC*, and *penDE*) are clustered together among other ORFs in a 56.8 kb region [10], [11]. Tandem duplications of this cluster can be found in *P. chrysogenum* strains that are high penicillin producers [12]. Other enzymes outside the core cluster are required to activate the first step in the pathway as well as to activate the side chains [13]. The last two steps in the penicillin biosynthesis pathway take place in the microbody (peroxisome), and strains with more microbodies produce more penicillin [14]. Adding precursors such as penylacetic acid (PAA) to the culture medium pushes synthesis towards penicillin G, one of two main commercial penicillins [15]. The penicillin biosynthesis cluster appears devoid of regulators specific to penicillin production [11], [16], and regulation of the process seems to be controlled by heterochromatin modification, nitrogen regulation, and pH-dependent carbon source regulation [7], [13].

Improvement for industrial production required selection not only for β-lactam synthesis but also for growth in submerged culture. Relative to NRRL 1951, improved strains have an increased ability to deal with oxidative stress, a reduced range of secondary metabolite production concomitant with an increase in penicillin output, and a decrease in the expression of proteins associated with virulence and cell wall degradation [17]. Other methods of increasing penicillin production include modifying the growth conditions and reducing sporulation and growth, which occur at the expense of secondary metabolite production [18].

The sequencing of one improved Wisconsin family strain (WIS 54–1255) has produced insights into the genetics of penicillin biosynthesis [16], but this information alone is insufficient to elucidate the genomic changes between wild strains, improved strains, and wild strains with enhanced penicillin production. The WIS 54–1255 strain (hereafter referred to as WI) was produced via multiple rounds of selection and mutagenesis, including ultraviolet radiation, X rays, and nitrogen mustard, which may have led to chromosomal rearrangements [3], [5], [6]. There is a previously identified inversion in the biosynthesis cluster [11], [12], presumably induced by strain improvement efforts.

We utilized Illumina short read sequencing of a wild *P. chrysogenum* strain (PC0184C, hereafter referred to as UCB) to develop an assembly-free computational pipeline using mate-pair information to identify chromosomal rearrangements between this wild strain and an improved strain (WIS 54–1255). We further validated our *in silico* predictions with a PCR-based assay and screened additional wild and industrially important *P. chrysogenum* strains to assess which, if any, genomic changes are specific to the industrially important strains and thus potentially contribute to their improved capacity for penicillin biosynthesis.

## Results

### Chromosomal rearrangements between the wild UCB strain of *P. chrysogenum and the industrial WI strain*

**Figure 1:**
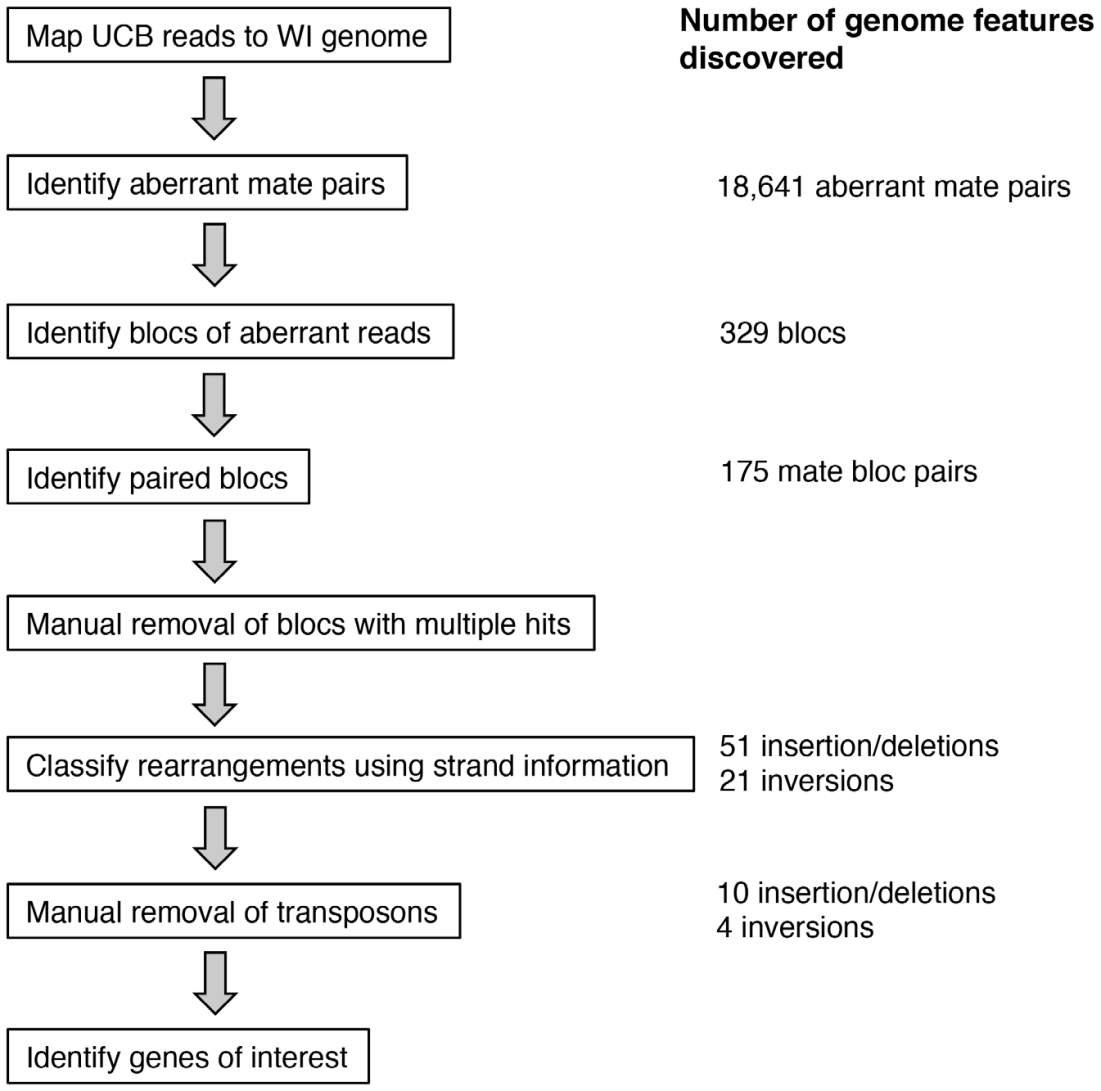
Flow chart for assembly-free rearrangement identification. Steps for identifying, classifying, and validating rearrangements. The number of genome features discovered at each stage are noted.

**Figure 2:**
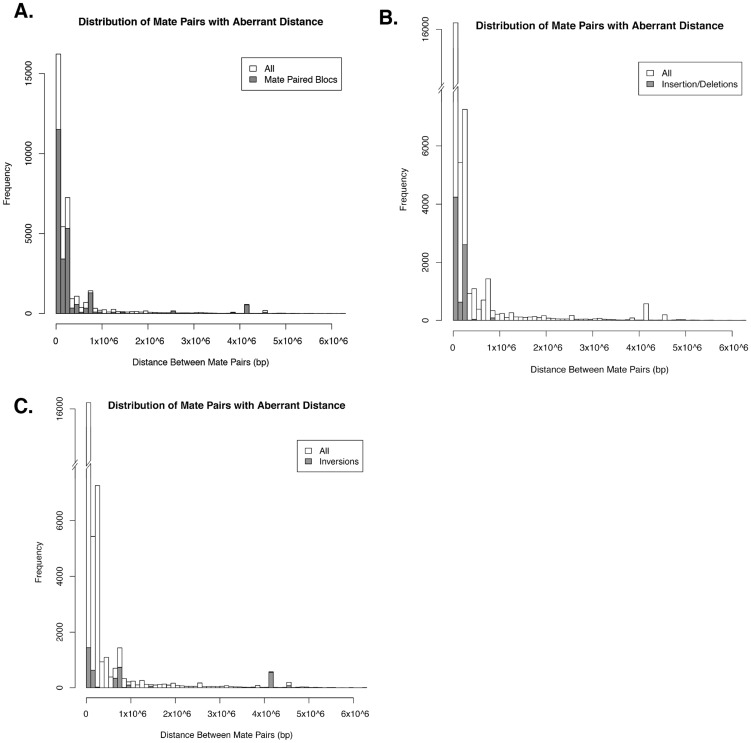
Distribution of mate pairs. Distribution of all aberrant mate pairs by distance (white) overlaid with mate pairs in all mated blocs (**A.**), mate pairs in blocs defining putative insertion/deletions (**B.**), and mate pairs in blocs defining putative inversions (**C.**).

**Figure 3:**
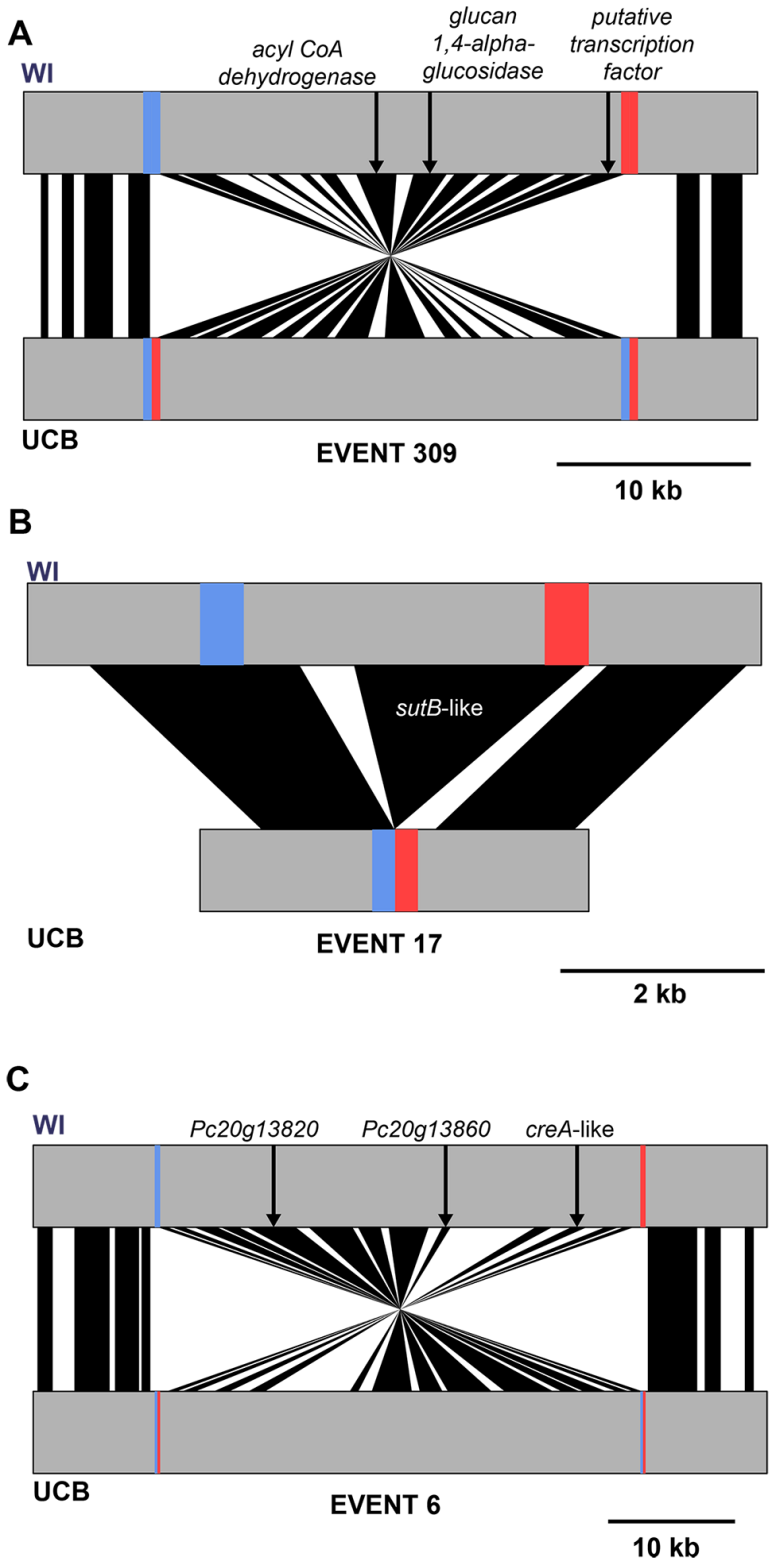
Architecture of rearrangements between the WI strain and the wild UCB strain. Rearrangement events containing genes with potential roles in penicillin biosynthesis are shown with their predicted gene contents (black spans). Flanking blocs of aberrantly mapping reads are marked in blue and red and penicillin-related genes are denoted by arrows. For those genes with known function, the gene name is listed, otherwise the gene ID is given. (A) Event 309 is an inversion and contains the predicted glucan 1,4-alpha-glucosidase Pc13g11940 and the gene of unknown function Pc13g11930, both of which show elevated expression in high penicillin G producing strains [16], [20] as well as Pc13g11930, which is predicted to localize to the microbody where penicillin biosynthesis takes place. (B) Event 17 is an insertion of Pc12g01540, a gene with strong similarity to the sulfate transporter *sutB,* which also shows elevated expression in high penicillin-producing strains and may be involved in biosynthesis of amino acid β-lactam precursors [16], [20]. (C) Event 6 is an inversion containing three genes that are repressed in high penicillin-producing strains: Pc20g13820, Pc20g13860, and Pc20g13880 [16], the latter of which is a homolog of the *Aspergillus niger creA* regulator of β-lactam biosynthesis.

We carried out paired-end Illumina sequencing of genomic DNA of the wild UCB strain, mapped reads to the published WI genome, and used the mapping data to identify putative chromosomal rearrangements that differentiated this strain from the industrial Wisconsin strain (Fig. 1; see Methods for details). Briefly, we expected that rearrangement events would be detectable based on the mapping of mate pairs to locations in the WI genome assembly much further apart than the 300-500 bp fragment insert sizes used to prepare the library (Fig. 2). Reads from any rearrangement event should cluster together on opposite sides of the breakpoint positions, given the amplification using primers complementary to opposite strands of genomic DNA fragments during Illumina library construction [19]. An analysis pipeline based on this expectation (Fig. 1) located 51 candidate insertion/deletion events and 21 candidate inversion events. Manual inspection suggested that the origin of many of these candidate rearrangements likely lay in transposable element gains and losses (see Methods). Eliminating the latter from further consideration, we identified 10 large insertion/deletion events (>1 kb) and 4 inversions (Fig. 3 and Table 1) in gene-rich regions. These inferred events included the single previously described structural difference between WI and other wild strains, which lay in the penicillin biosynthesis gene cluster [12] (event 326 in Table 1).

**Table 1:** Summary of PCR validations of rearrangement events.

Event Type	Event	Size (bp)	WI DNA		UCB DNA	
			WI Config	UCB Config	WI Config	UCB Config
Inversion	6	37,858	+	–	–	+
Insertion/Deletion	12	1,905	+	–	–	+
Insertion/Deletion	17	2,956	+	–	–	+
Insertion/Deletion	231	1,954	*	–	–	+
Insertion/Deletion	237	3,777	+	–	–	+
Insertion/Deletion	267	1,857	+	–	–	+
Insertion/Deletion	269	649	+	–	–	+
Insertion/Deletion	270	5,892	+	–	–	+
Insertion/Deletion	287	2,059	+	–	–	+
Inversion	309	23,748	+	–	–	+
Inversion	312	4,135,317	+	–	–	+
Insertion/Deletion	317	683	+	–	–	+
Insertion/Deletion	318	5,891	+	–	–	+
Inversion	326	2,275	+	–	–	+

PCR products for each primer configuration were scored as present (+), absent (–), or ambiguous (*) based on their migration on a 1.5% agarose gel (Fig. S2). Present scores indicate very bright bands of the expected size for both of the blocs spanning each breakpoint of an event. Absent scores indicate the lack of a bright band of the expected size for one or both blocs. The ambiguous score indicates unclear results. Event sizes are the number of base pairs in the WI genome internal to two mate blocs.

**Figure 4:**
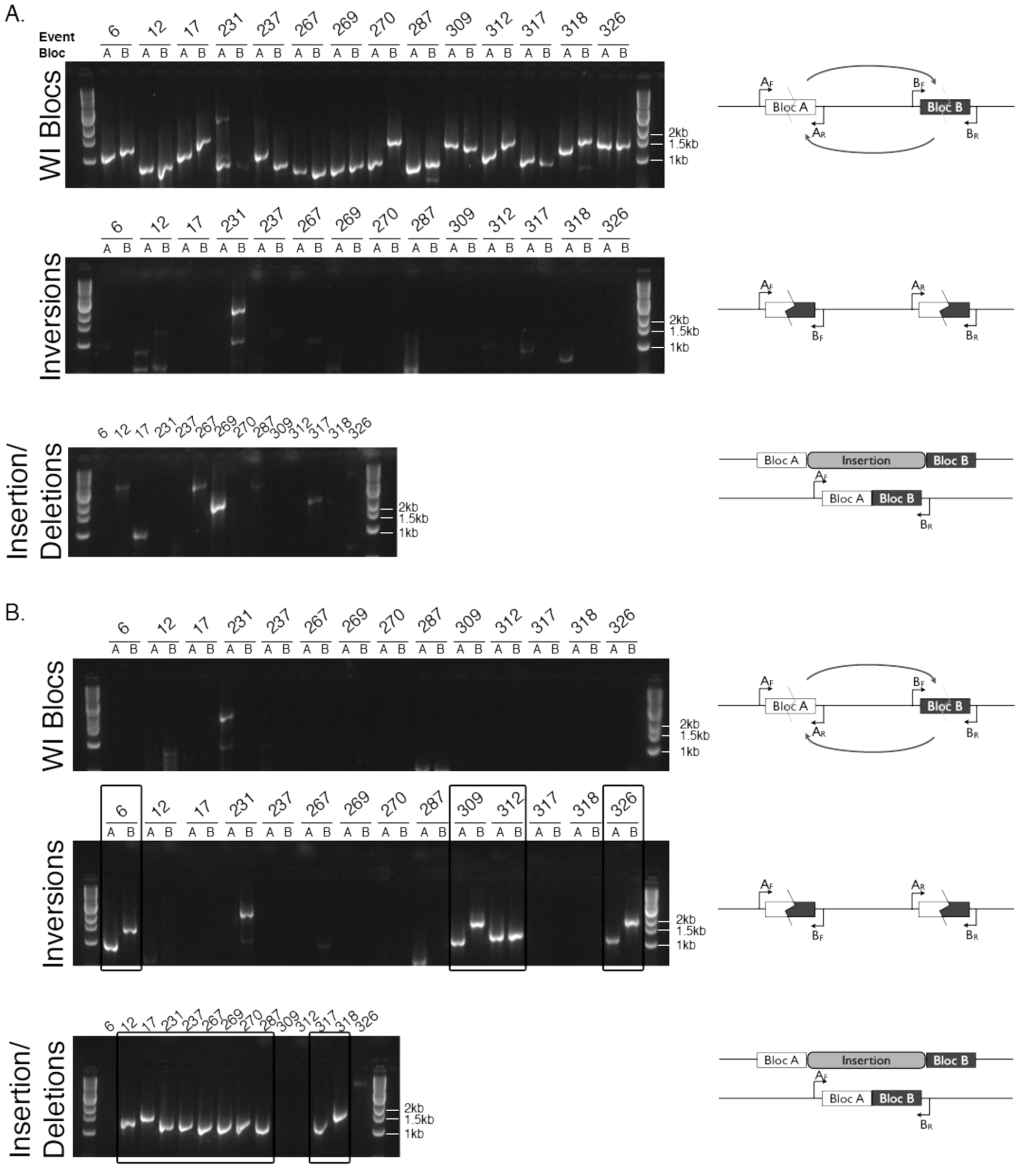
PCR validation of rearrangements identified *in silico*. PCR reactions used DNA from the **A.** WI strain and **B.** UCB strain. The top gel image in each figure used primers in the orientation to amplify across blocs in the WI strain. Middle images had primers to amplify across blocs in the UCB strain for a relative inversion. Bottom images show reactions priming across insertions in the WI strain relative to the UCB strain. Diagrams to the right of each gel image depict the primer configurations for PCR amplification across blocs. Small arrows indicate relative position and direction of forward (F) and reverse (R) primers for a set of paired mate blocs in the WI strain. Zigzag lines indicate breakpoints. Curved arrows indicate the direction of inversion. All PCR products for each strain were run on a single gel. Each PCR reaction is labeled by event and bloc (A or B), and boxes highlight successful amplification of rearrangement events in the UCB strain.

To validate each of these inferred rearrangements between the UCB and WI strains, we designed single-locus PCR-based assays (Fig. 4 and Table 1). Each amplification gave the product size expected from the event inferred *in silico*, with the exception of a likely complex rearrangement near the gene Pc22g09350 (Fig. 4). Sequencing of amplified products likewise confirmed the inferred events in each case (Table S1).

### Rearrangements between UCB and WI are positioned preferentially near penicillin-related genes

We hypothesized that many of the chromosomal rearrangements we identified between the WI and UCB strains could act in *cis* on penicillin-related genes to contribute to the high penicillin production observed in WI. As an unbiased test of this notion, we used a set of 522 genes with functions related to penicillin synthesis based on expression profiles from Harris et al. [20]. Excluding the previously characterized rearrangement at the penicillin biosynthesis cluster (event 326 in Table 1) to avoid potential bias towards known factors in high penicillin production, we found that 8 penicillin-related genes were also among the 96 genes residing within 5 kb of our PCR-verified rearrangement breakpoints, an enrichment beyond that expected by chance (Tables 3, S2, and S3) (hypergeometric *P* = 0.038).

Manual inspection of the genomic regions flanked by the rearrangement breakpoints between WI and UCB further supported the hypothesis that these structural changes influenced genes involved in penicillin biosynthesis. For example, a large inversion (event 309 in Table 1, Fig. 3, and Table S2) involved the predicted glucan 1,4-alpha-glucosidase Pc13g11940, highly expressed in a high penicillin G producer [16]; Pc13g11930, predicted to localize to the microbody where penicillin biosynthesis takes place [21]; and Pc13g11930, which is highly expressed in penicillin-producing strains [16], [20]. An insertion (event 17 in Fig. 3 and Tables S2 and S4) involved Pc12g01540, a gene with strong similarity to the sulfate transporter *sutB*, which likewise is highly expressed in high penicillin-producing strains and may be involved in biosynthesis of amino acid β-lactam precursors of penicillin [16], [20]. Another large inversion (event 6 in Table 1, Fig. 3, and Table S2) involved three genes repressed in high penicillin-producing strains, Pc20g13820, Pc20g13860, and Pc20g13880 [16], the latter of which is a homolog of the *Aspergillus niger creA* regulator of β-lactam biosynthesis. These findings establish a strong relationship between penicillin genes and structural rearrangements in the comparison of the WI industrial strain with the UCB wild isolate.

### Most genomic rearrangements present in WI are segregating in wild *P. chrysogenum*

We expected that if chromosomal rearrangements occurred while the progenitor of the WI strain was subjected to mutagenesis and selection for increased penicillin production, such structural changes should be specific to the WI genome. To test this, we used our PCR assays to determine the orientation of these regions in the wild progenitor of the WI strain, NRRL 1951. Surprisingly, for nearly all the rearrangements that distinguished UCB from WI, the latter resembled its wild progenitor (Table 2). Only at the previously characterized rearrangement at the penicillin biosynthesis gene cluster (event 326 in Table 2 and Fig. 4) did the WI strain differ from NRRL 1951. These results strongly suggested that the majority of differences in chromosome structure between the UCB and WI strains had not arisen recently during artificial selection in the industrial setting. Instead, we hypothesized that these rearrangements were ancient alleles segregating in wild *P. chrysogenum* populations.

**Table 2:** PCR validation results for all rearrangement events across several strains.

Event	WI	NRRL 1951	NRRL 832	NRRL 824	Henk PC08-3A	NRRL A3704	UCB
6	WI	WI	WI	UCB	UCB	WI	UCB
12	WI	WI	WI	UCB	UCB	UCB	UCB
17	WI	WI	WI	UCB	UCB	UCB	UCB
231	WI	WI	WI	WI	WI	UCB	UCB
237	WI	WI	WI	WI	WI	WI	UCB
267	WI	WI	*	WI	WI	UCB	UCB
269	WI	WI	UCB	UCB	UCB	UCB	UCB
270	WI	WI	UCB	UCB	UCB	UCB	UCB
287	WI	WI	WI	UCB	*	UCB	UCB
309	WI	WI	WI	WI	WI	WI	UCB
312	WI	WI	UCB	UCB	UCB	UCB	UCB
317	WI	WI	WI	UCB	WI	WI	UCB
318	WI	WI	UCB	UCB	UCB	UCB	UCB
326	WI	UCB	*	UCB	UCB	UCB	UCB

NRRL 1951 differed from its improved descendant, the WI strain, in the state of only event 326. The WI strain consistently showed the same ambiguous banding for event 231 (Fig.S2), and strains labeled WI for that event were ambiguous in the same manner. Cells are designated for WI conformation (WI), UCB conformation (UCB), or ambiguous banding (*). The WI strain, NRRL 1951, NRRL 832, and NRRL 824 are known penicillin producers.

**Table 3:** Genes involved in penicillin biosynthesis and associated with rearrangement breakpoints.

Gene ID	Annotation (from Harris *et. al.* 2009)	Event #
Pc12g00460	strong similarity to multidrug resistance protein fnx1p – *S. pombe*	12
Pc12g01540	strong similarity to sulfate permease SutB – *P. chrysogenum*	17
Pc18g03010	strong similarity to choline permease Hnm1 – *S. cerevisiae*	267
Pc18g03120	strong similarity to hypothetical protein mg00375.1 – *M. grisea*	269
Pc21g09650	weak similarity to ecto-ATPase c-cam105 – *R. norvegicus*	317
Pc21g13940	strong similarity to hypothetical protein An03g01270 – *A. niger*	318
Pc21g13950	strong similarity to aspergillopepsin II – *A. niger*	318
Pc22g09350	strong similarity to vacuolar H(+) Ca(2+) exchanger Vcx1 – *S. cerevisiae*	231

These genes are located within 5 kb of rearrangement breakpoints and have putative functions related to penicillin biosynthesis based on an expression profiling experiment performed by Harris et al (2009).

To assess structural variation across *P. chrysogenum* strains, we assayed the 14 rearrangements we had identified between UCB and WI (Table 1) in a panel of additional isolates: the original strain isolated by Fleming (NRRL 824); NRRL 832, identified as a high penicillin producer in submerged culture [6]; and two recently isolated wild strains (Henk PC08-3A and NRRL A3704). As predicted from our analyses of WI and UCB, almost all the rearrangements were polymorphic across this strain panel (Table 2), apart from the event at the penicillin gene cluster (event 326 in Table 2). We posit that the rearrangements are polymorphic within a single *P. chrysogenum* population, given the evidence for globally recombining populations [22]. Taken together, our results make clear that chromosomal rearrangements are widespread among *P. chrysogenum* strains, and they fall preferentially in regions proximal to genes involved in penicillin biosynthesis.

## Discussion

How to maximize production of commercially relevant biomolecules is one of the central questions in industrial microbiology. For the vast majority of industrial strains producing any given molecule, the genetic basis of the production trait and its potential for further improvement remain unknown. In this work, we have identified structural rearrangements in a strain of *P. chrysogenum* used for decades in industrial penicillin production. We have shown that most of these genomic changes have the potential to affect penicillin biosynthesis yet are unlikely to be the product of artificial selection. The one exception, a rearrangement at the penicillin biosynthesis gene cluster, is known to distinguish the WI family of strains from wild isolates of *P. chrysogenum* [12], supporting a model in which this mutation was indeed a product of the strain improvement process. The fact that structural changes at this locus have not been observed in wild strains suggests the presence of strong purifying selection outside of the industrial setting.

Apart from structural changes at the penicillin biosynthesis gene cluster, our findings suggest that NRRL 1951, the progenitor of the WI strain and of many others used in industrial production, naturally inherited genomic attributes that predisposed it toward high penicillin yield. Of the many wild strains that have been screened for penicillin production, NRRL 1951 was among a very few chosen for improvement. Our findings raise the possibility that additional determinants of penicillin production are segregating among wild *P. chrysogenum* strains. We speculate that more extensive surveys of wild isolates for penicillin yield may enable industrial microbiologists to take full advantage of the variation already present in nature.

## Materials and Methods

### Ethics Statement

No human or animal subjects were used in the course of this work.

### Cultures and Strains

The following *P. chrysogenum* strains were obtained from the ATCC culture collection: 28089 (designation WIS 54–1255 and hereafter referred to as WI), 9480 (NRRL 1951), 9179 (NRRL 832), and 9478 (NRRL 824, originally deposited as *P. notatum*). NRRL 1951 is the founding member of the industrially important Wisconsin family of improved *P. chrysogenum* strains [6]. The WI strain is descended from NRRL 1951. ATCC 9179 was identified as a strain particularly suited for penicillin production in submerged culture, which is preferable to surface culture for industrial production [23]. ATCC 9478 is Fleming's original strain [1].

The following wild strains were graciously provided by Daniel Henk (Imperial College, London): PC0184C, PC081B, PC083A, PC085A, PC088B, PC0820A, PC0897A, NRRL A1723, NRRL A2416, NRRL A24700, NRRL A3704, NRRL 1975, NRRL A3704, NRRL A3905, NRRL A20320, NRRL 32232, 924700, and PC0887A. We sequenced PC0814C, which was collected by Dr. Mark Enright (Imperial College) in January 2008 from Morzine, France. The strain was isolated from an adhesive film exposed to the air for 6 hours [22]. For brevity we refer to this strain as UCB. All *P. chrysogenum* strains were maintained on MEA (20 g/L malt extract, 1.5% agar) at room temperature.

### UCB Strain (PC0814C) Sequencing

The UCB strain was sequenced with three lanes of paired-end Illumina (San Diego, CA) 36 bp reads, following standard Illumina protocols, at the Vincent J. Coates Genomic Sequencing Laboratory (UC Berkeley, Berkeley, CA). Insert sizes were 300 bp and 500 bp. Sequence reads were submitted to the NCBI Sequence Read Archive (accession number SRP040942).

### Read Mapping

We mapped reads to the published *P. chrysogenum* WI genome assembly [3] using Bowtie [24], discarding all alignments for reads that mapped equally well to multiple places in the genome. To ensure that mate pairs with larger than expected mapping distances were not discarded during the alignment process, we disregarded mate pair information while mapping. We then identified the location of mate pairs in the WI genome assembly post-alignment using mate-pair information encoded in the Bowtie mapping output. After pairing mates together, the distance between their genomic locations was calculated, identifying 18,641 aberrant mate pairs on the same contigs with distances over 1,000 bp. The distances between aberrant mates that mapped to the same contig were not evenly distributed, with most mate pairs being either less than 1 Mb apart or just over 4 Mb apart (Fig. 2). These aberrant mate pairs formed 329 blocs of at least 20 reads with start positions no more than 100 bp from each other. These blocs were further narrowed down to 175 pairs of mate blocs by taking the median mate pair distance for reads within a bloc and searching for other blocs within 1 kb of that distance. Mate paired blocs were manually curated to correct for instances where one bloc had multiple hits.

### Support for Rearrangements from High Coverage

Because the UCB strain genome was sequenced at ∼30X coverage, each rearrangement breakpoint in the WI genome should be spanned by ∼30 different mate pairs from the UCB reads. These pairs (presumably located within 500 bp of each other in the UCB genome) should map to locations in the WI genome at distances equal to the size of the rearrangement event (Fig. 2). We required read clusters or “blocs” to be composed of at least 20 reads from aberrant mate pairs located no more than 100 bp from each other. We also identified “mate blocs” by pairing up blocs that had at least 90% of the reads mate paired together. A pair of mate blocs therefore represents the boundaries of a single putative rearrangement event. Mate blocs were further manually curated to filter out putative repetitive elements.

### Using Strand Information to Classify Rearrangements

Strandedness of aberrant mate pairs that map to the same contig was used to classify rearrangements as either inversions or insertions/deletions. An insertion event in the WI strain or a deletion in the UCB strain would result in mate pairs mapping to opposite strands, preserving the mate pair directions. An inversion event would flip the strand of one read in each mate pair, resulting in mate pairs mapping to the same strand. Events classified as insertions were required to be supported by at least 90% of aberrant mate pairs mapping to opposite strands, while inversion events were required to be supported by at least 90% of aberrant mate pairs mapping to the same strand.

### PCR Validation

In order to validate the rearrangement events predicted *in silico*, we designed primers to amplify across blocs as arranged in the WI strain (Fig. 3). If the predicted rearrangement were correct, using the primers in the same orientation with DNA from the UCB strain, no product should be detected. However, if PCR product amplified when we switched the primer pairs to be either both forward or both reverse primers from a mate bloc pair, this was consistent with an inversion event. Insertions were detected by priming across the insertion, using a forward primer for one bloc in a mate bloc pair and the reverse primer from its mate. PCR products for the WI and UCB strains were further validated by sequencing at the UC Berkeley Sequencing Facility.

### DNA Extraction

Prior to extraction, all *P. chrysogenum* cultures were grown in flasks containing 100 ml liquid malt extract medium (20 g/L malt extract) for two days at 25°C with gentle shaking. Tissue was harvested by filtering through Miracloth (Calbiochem, Darmstadt, Germany) and rinsing with ∼100 mL sterile distilled water. Samples were frozen in liquid nitrogen and stored at −80°C prior to lyophilization for 2 days. DNA was extracted by beadbeating with 0.3 g zirconia/silica beads (BioSpec Products, Bartlesville, OK) in a screwtop tube for 30 sec. Following addition of 0.5 mL lysis buffer (50 mM Tris-HCl, 50 mM EDTA, 3% SDS, and 1% β-mercaptoethanol), tubes were vortexed to resuspend all ground tissue and then incubated at 65°C for 45 min. Chloroform (0.5 mL) was added, and tubes were vortexed and then spun at 1,320 rpm for 5 min. The aqueous phase (∼350 µl) was transferred to a new tube with 35 µl Proteinase K and 350 µl Buffer AL from the DNeasy Blood and Tissue kit (Qiagen, Valencia, CA), and manufacturer's directions were subsequently followed.

### Primer Design and PCR

Primers were designed with the PrimerQuest^SM^ tool by Integrated DNA Technologies (IDT) to amplify across each bloc in the WI-54-1255 genome (Table S1). Various primer pairs were selected to identify blocs in the WI arrangement, blocs that were inverted relative to WI, and blocs with insertions relative to WI. Each 25 µl reaction was made according to the following recipe: 1 µl of DNA extract, 2.5 µl of 2 mM dNTPs (Fermentas, Glen Burnie, MD), 2.5 µl buffer (0.5 M KCl, 0.1 M TrisHCl pH 8.3, 25 mM MgCl_2_, and 1 mg/mL gelatin), 0.25 µl of each primer (50 µM, IDT, San Diego, CA), 0.5 µl Taq DNA polymerase (New England Biolabs, Ipswitch, MA), and 18 µl water. Reactions were run with the following PCR program:

1. 94°C for 1 min
2. 94°C for 1 min
3. 68°C for 1 min
4. 72°C for 1.5 min
5. Repeat steps 2 through 4 30 times.
6. 72°C for 8 min

*P. chrysogenum* WI-54-1255 Genes Associated with Rearrangements

Genes of interest were identified as those whose start was either within a rearrangement event or less then 500 bp outside it. The very large rearrangements were ignored for this purpose due to the sheer number of genes involved.

## Supporting Information

Table S1 **Primer sequences designed to amplify across Wisconsin blocs.** Each primer was named based on the target bloc and the forward (F) or reverse (R) direction of priming.
(DOCX)

Table S2 **Genes associated with validated rearrangement events and found in the literature.** Genes of interest were identified as those whose start was either within a rearrangement event or less than 500 bp outside it. The very large rearrangements were ignored for this purpose due to the sheer number of genes involved. Genes were annotated by van den Berg et al.
(DOCX)

Table S3 **Genes associated with validated rearrangement events and annotated by van Den Berg et al. but otherwise undescribed.** Genes of interest were identified as those whose start was either within a rearrangement event or less then 500 bp outside it. The very large rearrangements were ignored for this purpose due to the sheer number of genes involved. Interpro terms were not available (NA) for all annotations.
(DOCX)

Table S4 **Contents of insertion events.**
(DOCX)
