# Supplementary material for: Structural Variation among Wild and Industrial Strains of Penicillium chrysogenum
Source: PLoS One. 2014 May 13;9(5):e96784. doi: 10.1371/journal.pone.0096784 (PMC4019546; doi:10.1371/journal.pone.0096784)
Supplement: Table S1 — Primer sequences designed to amplify across Wisconsin blocs. Each primer was named based on the target bloc and the forward (F) or reverse (R) direction of priming. (DOCX) [file pone.0096784.s001.docx]

**Table S1. Primer sequences designed to amplify across Wisconsin blocs.**

| **Primer Name** | **Sequence** |
| --- | --- |
| Bloc_6A_F | CTCGCATGCATTGGGCTTATCGTT |
| Bloc_6A_R | AGTCTCCTCAACCAACACCAACCA |
| Bloc_6B_F | TGCCTACTTGTTGTGCCTAGGTGT |
| Bloc_6B_R | TTAACGCCTACCTCGCTGCTGTTA |
| Bloc_12A_F | TGCAGAGGGTGATGCAAGACTACA |
| Bloc_12A_R | AGATCCTCATATTTCGCACGCTCG |
| Bloc_12B_F | AAGCCAGGACATAGGAGAGAGACA |
| Bloc_12B_R | GCACAAGTCTTACATCACTGGGCT |
| Bloc_17A_F | ATCAACTTGGCCTGGTCTCTTCCA |
| Bloc_17A_R | GGGAAACTGAAGCCCACAAATGGT |
| Bloc_17B_F | TGATCGTTCCACCCAACACGATCT |
| Bloc_17B_R | GTTGTTGTCTCCCATGCGCTTGAA |
| Bloc_231A_F | AACAAGGAATCCACCGAGACTCCA |
| Bloc_231A_R | TAGTCCTCCTTCGTAGCCTCAACA |
| Bloc_231B_F | AGGCGGTAGGATTAGTGCCTGATT |
| Bloc_231B_R | AGGTACCTGTCTCTGGTAGCGAAT |
| Bloc_237A_F | AGTGATGTGGATGGATCGGAGCAT |
| Bloc_237A_R | AACTACACCAAGTCCAGCACCAGT |
| Bloc_237B_F | AGGCGGTACTCATTAAGGCATCCA |
| Bloc_237B_R | CTCACGTGGCTGACACAAAGTCAA |
| Bloc_267A_F | AGCTCCAAGTTACCGGTTTCTCCT |
| Bloc_267A_R | GAAGATTCGCCATTTCTCGCACCA |
| Bloc_267B_F | TGCAGGAAGCCAGGATATAGGAGA |
| Bloc_267B_R | TAGGCATCCTAGCGCTGTCGATTT |
| Bloc_269A_F | ACAACTTGTTCGTATGAAGCCGGG |
| Bloc_269A_R | GCATGCATTCGTATGGGAGTAAATGGC |
| Bloc_269B_F | AGAGAGCGGTCCTAATTCAGGCAT |
| Bloc_269B_R | TTGCCCTTGTCGCAGATCATCCTA |
| Bloc_270A_F | AACATGCCTTCAACAGTTCCACCG |
| Bloc_270A_R | TCGACATCCAGCTTATCGACAGCA |
| Bloc_270B_F | AAATCGGTTCGTTCTTTCAGCGGC |
| Bloc_270B_R | TCTCGAGAAACAAACCAGCATAGC |
| Bloc_287A_F | AGGGAGAGAAGAGCAGAAGAAGGT |
| Bloc_287A_R | CCCTACAAAGGCATGCTTCTACCTGA |
| Bloc_287B_F | AACCTGATGGCGGCAACGAGAATA |
| Bloc_287B_R | TATTCAAGCCTCTGGCACCAACCA |
| Bloc_309A_F | AGAGGATGCAAATCTCGGCCACTT |
| Bloc_309A_R | ATGCAGAGCCAGTGGTCACAATAC |
| Bloc_309B_F | TCTCGCGTAGGGCTGTTTGAAGAT |
| Bloc_309B_R | TGTGCGCATCCATTCGGAGTGTTA |
| Bloc_312A_F | AGACGATCTCGGAAACAACGCTGA |
| Bloc_312A_R | ACGCGACCAAGTACAAGAGAGCAA |
| Bloc_312B_F | AGTGAAGGGATCGAATGGAGGACA |
| Bloc_312B_R | GCTATCGCTTTGCAGCTTGCGTAT |
| Bloc_317A_F | TGTCTTTCCTAGCGCCATCGACAT |
| Bloc_317A_R | ACCGACATCACTCTGCTGGTGAAA |
| Bloc_317B_F | ACCAGCAGAGTGATGTCGGTTTCT |
| Bloc_317B_R | TGGACCTTCATGATGGGATGGGAA |
| Bloc_318A_F | GAAATGACAGCGGCACAACGGTTA |
| Bloc_318A_R | TTTGTACGGACAGCAACAATGCCG |
| Bloc_318B_F | AGTCTCGGTTTGCAGCAGGTTAGT |
| Bloc_318B_R | TTTATCCGCGCTTGCGATGACTTG |
| Bloc_326A_F | ACCCTTGTGAGATACCAAAGTGCG |
| Bloc_326A_R | ACTGCTGCGTTAAGCGGACATCTA |
| Bloc_326B_F | TTTCGTGATGGAGGACACTTGGCA |
| Bloc_326B_R | TTCCTTGTAACCCAGTGGAGAGAG |

Each primer was named based on the target bloc and the forward (F) or reverse (R) direction of priming.
