# Supplementary material for: Structural Variation among Wild and Industrial Strains of Penicillium chrysogenum
Source: PLoS One. 2014 May 13;9(5):e96784. doi: 10.1371/journal.pone.0096784 (PMC4019546; doi:10.1371/journal.pone.0096784)
Supplement: Table S2 — Genes associated with validated rearrangement events and found in the literature. Genes of interest were identified as those whose start was either within a rearrangement event or less than 500 bp outside it. The very large rearrangements were ignored for this purpose due to the sheer number of genes involved. Genes were annotated by van den Berg et al. (DOCX) [file pone.0096784.s002.docx]

**Table S2. Genes associated with validated rearrangement events and found in the literature.**

| **Name** | **Description** | **Rearrangement Event** |
| --- | --- | --- |
| Pc12g01540 | Strong similarity to sulfate_permease_sutB | 17 |
| Pc13g11930 | strong similarity to acyl CoA dehydrogenase aidB - *Escherichia coli* | 309 |
| Pc13g11940 | Glucan 1,4-alpha-glucosidase glaA | 309 |
| Pc13g11990 | Putative transcription factor CAF32051.1 | 309 |
| Pc20g13820 | Strong similarity to hypoProt_An02g03740 | 6 |
| Pc20g13860 | Strong similarity to hypoProt_An02g03790 | 6 |
| Pc20g13880 | Strong similarity to CreA; TF involved in β-lactam biosynthesis; Glucose repressor | 6 |
| Pc20g13890 | Duplicate or misannotation of creA | 6 |
| Pc21g21270^*^ | Strong similarity to hypothetical protein B9I2.20 - *Neurospora crassa* | 326 |
| Pc21g21280^*^ | Strong similarity to methyl sterol oxidase Erg25 - *Saccharomyces cerevisiae* | 326 |
| Pc21g21290^*^ | Strong similarity to hypothetical protein mg02069.1 - *Magnaporthe grisea* | 326 |

Genes of interest were identified as those whose start was either within a rearrangement event or less than 500 bp outside it. The very large rearrangements were ignored for this purpose due to the sheer number of genes involved. Genes were annotated by van den Berg et al.
