# Supplementary material for: Structural Variation among Wild and Industrial Strains of Penicillium chrysogenum
Source: PLoS One. 2014 May 13;9(5):e96784. doi: 10.1371/journal.pone.0096784 (PMC4019546; doi:10.1371/journal.pone.0096784)
Supplement: Table S3 — Genes associated with validated rearrangement events and annotated by van Den Berg et al. but otherwise undescribed. Genes of interest were identified as those whose start was either within a rearrangement event or less then 500 bp outside it. The very large rearrangements were ignored for this purpose due to the sheer number of genes involved. Interpro terms were not available (NA) for all annotations. (DOCX) [file pone.0096784.s003.docx]

**Table S3. Genes associated with validated rearrangement events and annotated by van Den Berg et al. but otherwise undescribed.**

| **Name** | **Interpro Terms** | **Rearrangement Event** |
| --- | --- | --- |
| Pc13g11880 | Tubulin/FtsZ, 2-layer sandwich domain;Tubulin, conserved site;Gamma tubulin; Tubulin/FtsZ, C-terminal; Tubulin/FtsZ, GTPase;Tubulin | 309 |
| Pc13g11890 | Zinc finger, AN1-type | 309 |
| Pc13g11950 | Phosphotyrosyl phosphatase activator, PTPA | 309 |
| Pc13g11970 | Actin/actin-like | 309 |
| Pc13g11980 | NAD(P)-binding;6-phosphogluconate dehydrogenase, NAD-binding;Dehydrogenase, multihelical;6-phosphogluconate dehydrogenase, C-terminal-like | 309 |
| Pc13g12000 | GTP cyclohydrolase II | 309 |
| Pc21g13970 | Fumarate reductase/succinate dehydrogenase flavoprotein, N-terminal | 318 |
| Pc21g13990 | Monooxygenase, FAD-binding;Aromatic-ring hydroxylase-like | 318 |
| Pc20g13780 | Histidine triad-like motif;Histidine triad motif | 6 |
| Pc20g13800 | Mob1/phocein | 6 |
| Pc20g13820 | Tyrosine protein kinase;Protein kinase, core | 6 |
| Pc20g13830 | Peptidase S16, lon C-terminal;Peptidase S16, ATP-dependent protease La;Peptidase S16, active site;Peptidase S16, Lon protease, C-terminal region;Peptidase S16, lon N-terminal;ATPase, AAA+ type, core;ATPase, AAA-type, core | 6 |
| Pc20g13840 | Chaperone DnaJ, C-terminal;Heat shock protein DnaJ, N-terminal;HSP40/DnaJ peptide-binding;Heat shock protein DnaJ;Heat shock protein DnaJ, cysteine-rich region;Heat shock protein DnaJ, conserved site | 6 |
| Pc20g13910 | Armadillo-type fold | 6 |
| Pc12g01550 | NA | 17 |
| Pc13g11870 | NA | 309 |
| Pc13g11900 | NA | 309 |
| Pc13g11910 | NA | 309 |
| Pc13g11920 | NA | 309 |
| Pc13g11960 | NA | 309 |
| Pc20g13790 | NA | 6 |
| Pc20g13810 | NA | 6 |
| Pc20g13850 | NA | 6 |
| Pc20g13870 | NA | 6 |
| Pc20g13900 | NA | 6 |

Genes of interest were identified as those whose start was either within a rearrangement event or less then 500 bp outside it. The very large rearrangements were ignored for this purpose due to the sheer number of genes involved. Interpro terms were not available (NA) for all annotations.
