# Supplementary material for: Structural Variation among Wild and Industrial Strains of Penicillium chrysogenum
Source: PLoS One. 2014 May 13;9(5):e96784. doi: 10.1371/journal.pone.0096784 (PMC4019546; doi:10.1371/journal.pone.0096784)
Supplement: Table S4 — Contents of insertion events. (DOCX) [file pone.0096784.s004.docx]

Table S4. Contents of insertion events.

| **Event** | **Contents** |
| --- | --- |
| 12 | MarinerTE |
| 17 | sulfate permease/sulfate transporter |
| 237 | conserved hypothetical protein repetitive within WI genome |
| 267 | Mariner/pogoTE |
| 269 | Repetitive Within WI genome; no other hits |
| 270 | Copia_like_retrotransposon |
| 287 | FlankedByMicrsatSeq_noMatchesInBLASTX_or_BLASTN |
| 317 | non-repetitive_noMatchesInBLASTX_or_BLASTN |
| 318 | CopiaTE |
